# Antimalarial drug resistance in *Plasmodium falciparum* isolates from the Pacific Coast of Colombia

**DOI:** 10.1017/S0031182026101711

**Published:** 2026-04

**Authors:** Kyle Michie, Bernice Chiu, Carla Briggs, Vladimir Corredor, Lorena Matta-Cortés, Julian C Rayner, Diego F. Echeverri

**Affiliations:** 1 Cambridge Institute for Medical Research (CIMR), University of Cambridge, Cambridge, UK; 2 Departamento de Salud Pública, Facultad de Medicina, Universidad Nacional de Colombia, Bogotá, Colombia; 3 Internal Medicine Department, Faculty of Health, Universidad del Valle, Cali, Colombia; 4 Departamento de Microbiología, Universidad del Valle, Facultad de Salud, Cali, Colombia

**Keywords:** antimalarial, *Plasmodium falciparum*, resistance, South America

## Abstract

Antimalarial drug resistance has evolved repeatedly and independently in both Southeast Asia and South America, but functional studies of parasite resistance have almost all concentrated on Asian isolates. Colombia contributes nearly one-third of all *Plasmodium falciparum* cases in South America, primarily focused on the Pacific Coast. We assessed the presence of resistance associated genotypes and phenotypes in this region using a panel of *P. falciparum* isolates collected across 23 years, with a specific focus on sensitivity to historically used antimalarials chloroquine, mefloquine, pyrimethamine and sulfadoxine. The sensitivity profiles were predicted genetically using a combination of quantitative PCR assays and sequencing of known resistance-associated loci, including the artemisinin resistance-associated gene PfKelch13. The isolates were then assessed phenotypically by introduction to *in vitro* culture allowing both antimalarial sensitivity testing and the establishment of a biobank of Colombian isolates for further work. We established that multiple antimalarial resistance associated genotypes and phenotypes, particularly for chloroquine and mefloquine, persist across the Colombian Pacific Coast but found no evidence for the presence of artemisinin resistance-associated polymorphisms. The continued presence of resistance against historically used antimalarials argues against the use of these drugs as partner therapy for artemisinin and highlights the unique epidemiological environment of the Pacific Coast which allows for long-term maintenance of resistance.

## Graphical abstract

**Figure:**
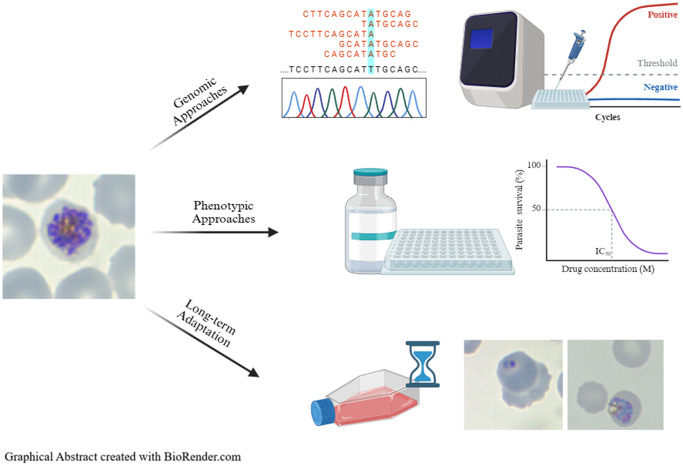

## Introduction

Malaria is a parasitic disease of global importance. Although the majority of the burden of the most pathogenic species, *Plasmodium falciparum*, lies in sub-Saharan Africa, there is an overlooked burden in South America, where close to one-third of *P. falciparumP. falciparum* cases occur in Colombia (World Health Organisation, 2024). The Pacific Coast of Colombia is of particular significance, where the population is predominantly Afro-Colombian and so largely resistant to *P. vivax* infection which dominates other regions of the country (González *et al.,* 2004). An increasing number of malaria cases in recent years (Instituto Nacional de Salud de Colombia, 2025) has meant that Colombia is on track to miss WHO targets in malaria control and reduction by 2025 (World Health Organisation, 2024).

The population of *P. falciparum* parasites in Colombia is shaped by the relatively low transmission rates, resulting in low complexity of infection, high introgression and linkage disequilibrium, which leads to low genetic diversity and the emergence of distinct, co-existing, clonal lineages. The lack of significant outcrossing means that these lineages are maintained over long periods of time to create a metapopulation structure (Ariey *et al.,* 2003; Echeverry *et al.,* 2013; Knudson *et al.,* 2020; Carrasquilla *et al.,* 2022). Each subpopulation is vulnerable to the effects of genetic drift and consequently there are high levels of variation between subpopulations but low variation within subpopulations (Anderson *et al.,* 2000; Ariey *et al.,* 2003; Feged-Rivadeneira *et al.,* 2018). These unique epidemiological dynamics have been demonstrated theoretically to accelerate the emergence of antimalarial resistance (Dye and Williams, 1997) and this has been borne out by the repeated emergence of antimalarial resistance in low-transmission areas of the Colombia–Venezuela region (Moore and Lanier, 1961; McCollum *et al.,* 2007).

Chloroquine resistance emerged independently in Colombia in the 1960s (Moore and Lanier, 1961) and is underpinned genetically by polymorphisms in PfCRT (Fidock *et al.,* 2000) and PfMDR1 (Reed *et al.,* 2000). The major PfCRT haplotype in the Pacific Coast region is CVM**ET** (positions 72–76, mutant amino acids in bold), which has historically been reported to be widespread in Colombia (Fidock *et al.,* 2000; Restrepo-Pineda *et al.,* 2008). A second PfCRT haplotype, **S**VM**NT**, emerged independently and is maintained in other regions of South America (Wootton *et al.,* 2002; Mita *et al.,* 2009; de Abreu-fernandes *et al.,* 2025). Whereas only 1 chloroquine-resistant PfCRT haplotype has been reported in Colombia, 2 major PfMDR1 haplotypes have been reported (Echeverry *et al.,* 2007; Montenegro *et al.,* 2021), N**F**S**D**D and N**F**S**DY** (positions 86, 184, 1034, 1042 and 1246, mutant amino acids in bold). These same polymorphisms in PfMDR1, along with increased copy number, mediate mefloquine resistance (Reed *et al.,* 2000; Price *et al.,* 2004). In Colombia, studies have shown an increasing prevalence of PfMDR1 amplification (Echeverry, 2013 Aponte *et al.,* 2017; Montenegro *et al.,* 2021), and there is further evidence of *in vivo* resistance (Carmona-Fonseca, 2004). Sulfadoxine–pyrimethamine (S-P) was used extensively in Colombia, particularly after chloroquine resistance emerged, and by 1981 there were reports of S-P resistance in Colombia (Espinal *et al.,* 1981; Carrasquilla *et al.,* 2022). Sulfadoxine resistance is associated to polymorphisms in PfDHPS (positions 431, 436, 437, 540, 581 and 613, ancestral haplotype ISAKAA (Triglia *et al.,* 1997; Sutherland *et al.,* 2009)) with genomic studies in Colombia showing a heterogenous pattern of resistance with exclusively ancestral haplotypes in southwest Colombia and the predominance of triple-mutant PfDHPS (IS**GEG**A, mutant amino acids in bold) in the Amazonía region (Corredor *et al.,* 2010). Pyrimethamine resistance is associated with polymorphisms in PfDHFR (positions 51, 59, 108 and 164, ancestral haplotype NCSI (Sirawaraporn *et al.,* 1997)) and genomic studies showed a similarly distributed, heterogenous pattern of resistance in Colombia (Corredor *et al.,* 2010). These patterns have been recapitulated by clinical efficacy studies (Osorio *et al.,* 2007; Gonzalez *et al.,* 2012). Lastly, amodiaquine was used in combination with S-P (Carmona-Fonseca, 2004), and there were similarly reports of the development of clinical resistance (Blair-Trujillo *et al.,* 2002; Blair *et al.,* 2008).

In 2006, due to the development of resistance to chloroquine, S-P and amodiaquine, artemisinin combination therapies (ACTs) became the front-line antimalarial in Colombia. There have been no artemisinin-resistance associated mutations thus far reported in Colombia (Aponte *et al.,* 2017), despite artemisinin-resistance associated haplotypes emerging elsewhere in South America (Mathieu *et al.,* 2020). ACTs appear to remain efficacious in the country (Aponte *et al.,* 2017; Montenegro *et al.,* 2021) despite the emergence of clinical resistance globally (Noedl *et al.,* 2008; Uwimana *et al.,* 2021). Given the historical precedence of the *de novo* emergence of resistance in Colombia, it is prescient to carefully monitor the landscape of antimalarial sensitivity in this region. While a small number genomic studies have given an overall picture of the landscape of resistant haplotypes in Colombia (Echeverry *et al.,* 2007; Restrepo-Pineda *et al.,* 2008; Corredor *et al.,* 2010; Aponte *et al.,* 2011, 2017; Echeverry, 2013; Montenegro *et al.,* 2021), there is no ongoing genetic surveillance. In addition, there is no established biobank of *in vitro* adapted Colombian *P. falciparum* isolates that can be used for sensitivity testing, and therefore it is not possible to verify the effects of putative resistance-mediating polymorphisms on the Colombian *P. falciparum* genomic backgrounds. We aimed to fill this gap by profiling both historical and contemporary *P. falciparum* isolates collected on the Pacific Coast of Colombia for antimalarial resistance genotypes and phenotypes. We further adapted some of these isolates into *in vitro* culture for future investigations into their antimalarial sensitivities and as a source for subsequent CRISPR editing to verify the potential effect of polymorphisms were they to arise or spread to the Colombian *P. falciparum* genomic background.

## Materials and methods

### Isolate collection

*Plasmodium falciparum* was cultured as previously described (Trager and Jensen, 1976). *P. falciparum* isolates were named using a 3-letter country code (Col for Colombia), followed by a 2-letter code for the city of origin (Gu for Guapi, Qu for Quibdo, Bu for Buenaventura, Tu for Tumaco, Ba for Bajo Baudo, and Ja for Jamundi), a 2-number code for the year (ranging from 99–23) and a numerical value to discriminate between isolates collected from the same region in the same year.

Historical isolates from 2000 to 2005 across the Colombian Pacific Coast were collected and adapted to *in vitro* human-serum supplemented culture (Echeverry *et al.,* 2006; Aponte *et al.,* 2011), and then in 2022, adapted to 0.5% (w/v) synthetic serum (AlbuMax2 – 11021037) – supplemented culture. Historical isolate ColGu03.04 was adapted to human-serum supplemented culture only, in 2023.

Contemporary isolates were collected from the *Hospital Universitario del Valle (HUV)* in Cali, from patient blood samples collected <24 h prior and maintained at room temperature, under ethical approval 023-023 from the *Universidad del Valle* and 02-2023 from *Hospital Universitario del Valle*. Samples were washed in RPMI, and subsequently cryopreserved or introduced directly to culture supplemented with both human and synthetic serum.

### Genotyping

Historical isolates had been whole genome sequenced previously (Echeverry *et al.,* 2013; MalariaGEN *et al.,* 2021), whereas loci of interest from contemporary isolates and historical isolate ColGu03.04 were sequenced as per below.

Genomic DNA from lysed and washed parasitized erythrocytes was extracted using either GeneJet Genomic DNA Purification Kit or Monarch Genome Extraction Kit, both modified to include 2 elution steps.

Regions of genes containing nucleotide polymorphisms associated to drug resistance were amplified, purified (using K0721, Thermo Fisher in Colombia, and T3010, NEBio in the United Kingdom) and sequenced (GeneWiz). Oligonucleotides for amplification and sequencing (Merck) were designed using reference gene sequences (PlasmoDB, https://plasmodb.org/plasmo/app) downloaded onto Benchling software. Regions of PfDHPS were amplified using ‘CCATCAGATGTTTATATAACAAATATGTGAGTAGG; and CG TCATGAACTCTTATTAGATCTACCTTTTTATAATAG’; whilst regions of PfDHFR were amplified using ‘GAACAAGTCTGCGACGTTTTCG; and CATCACATTCATATGTACTATTTATTCTAG’; and regions of PfMDR1 were amplified by nested PCR with sets of primers ‘GCATTTTATAATATGCATACTGTTATT and CAATGTTGCATCTTCTCTTC’; and ‘GCCTCTTCTATAATGGACATGG and ATGGGTAAAGAGCAGAAAGAGAAA’ (reverse primer from Lim *et al.,* 2013).

PfCRT was amplified and sequenced from purified genomic DNA using an established nested protocol (Chaijaroenkul *et al.,* 2011) and similarly for the propeller domain of PfKelch13, codons 440-693 were amplified as per (Ariey *et al.,* 2014).

### Copy number variation assays

For historical isolates, the purity of genomic DNA used for quantitative PCR was first assessed by absorbance ratios and minimum concentration using a NanoDrop Spectrophotometer, but the low *in vitro* parasitaemia of contemporary isolates and historical isolate ColGu03.04 meant less stringent thresholds were used. PfMDR1 copy number amplification was assessed using established primers (Lim *et al.,* 2013), and similarly with β-tubulin as a housekeeping gene (Eastman *et al.,* 2011). The protocol involved a Hot Start for the qPCR PowerUp Master Mix (A25918, Applied Biosystems) with a 2 min 50 °C and subsequent 2 min 95 °C incubation stage. The samples were then run for 39 cycles of 15 sec at 95 °C, 30 sec at 60 °C and 20 sec at 72 °C, followed by a melting curve analysis stage. Copy number was calculated using the Livak Method, using Excel, Microsoft; and the mean values plotted. Any copy number values <0.6 or out with >0.4 units from an integer were removed from subsequent analysis.

### Antimalarial sensitivity assays

*In vitro* antimalarial sensitivity assays were adapted from a protocol kindly provided by Professor Marcus Lee (University of Dundee). *Plasmodium falciparum* parasites were sorbitol synchronized, and after reinvasion, the culture was diluted to 0.5% parasitaemia and 1% haematocrit. Then, a 2-fold serial dilution of antimalarial drug was then added onto a 96-well plate, to which both the diluted parasitized erythrocytes, and unparasitized erythrocyte controls were added, with technical triplicates of each drug concentration. There were further controls of parasitized and unparasitized erythrocytes in the absence of drug. These assays were completed for chloroquine (C6628, Sigma-Aldrich), mefloquine (M2319, Sigma-Aldrich) and pyrimethamine (3918, Tocris). Two identical plates were completed on a given day for each isolate to form a single biological replicate. Where one plate failed to yield results, the remaining plate was used as the biological replicate. Different lab-adapted lines with established resistance profiles were used as controls, including 3D7 (Walliker *et al.,* 1987), 7G8 (Burkot *et al.,* 1984) and Dd2 (Wellems *et al.,* 1990). The plates were frozen and subsequently incubated in a 10 000X SYBR Green I Nucleic Acid Gel Stain (S7567, Invitrogen) with lysis buffer (20 mM Tris-HCl, 5 mM EDTA, 0.1% w/v saponin and 1% v/v Triton X-100 in MilliQ water) for >2 h at 37 °C, and read on a spectrophotometer with 485 nm excitation and 535 nm emission filters. The fluorescence values were exported to Excel, Microsoft.

Contemporary isolates and historical isolate ColGu03.04 were incubated on a shaking incubator prior to and during the antimalarial sensitivity assay. In Colombia, the cultures and assays plates were gassed using a custom gas mixture (1% O_2_/5% CO_2_/94% N_2_) and candle-jar technique, respectively. In Cambridge, the historical isolates were shaken prior to the assay only and incubated in 1% O_2_/3% CO_2_/96% N_2_. The use of the candle-jar technique contributed to a significant plate edge effect, and in these assays the first and final columns were removed from subsequent analysis to compensate for this. The short time of culture for contemporary isolates meant that at the time of the assay, they had not been successfully weaned off human-serum supplementation, and to avoid variability of different serum donors, the parasites were washed and assayed using synthetic serum-supplemented media. Isolate ColBa23.01 was assayed using an e*x vivo* approach, wherein parasites had completed only 1 growth cycle in culture media.

### Microscopy imaging

Giemsa stained thin smear slides were imaged using Carl Zeiss Axio Imager A2 microscope equipped with an AxioCam ERc5s digital camera (Carl Zeiss Microscopy, LLC, NY, USA) and further prepared using Zen 3.1 Microscope Software (Carl Zeiss Microscopy, Deutschland GmbH).

### Data analysis

The fluorescence values from the sensitivity screens results were normalized across each dilution series, and the subsequent triplicate values were plotted against drug concentration. A non-linear regression of a variable line was used to obtain the IC50 value. The IC50 values for each parasite isolate for a given antimalarial were subject to an outlier analysis using ROUT method (Motulsky and Brown, 2006) (>10%). The significance between a given isolate and negative control was calculated using a 1-way ANOVA with multiple comparisons to the negative control, assuming normal distribution and equal standard deviation, and Dunnet’s multiple comparisons test.

Comparison between groups of isolates, following outlier analysis, was carried out using Mann–Whitney *U*-test.

Means, standard deviations and all statistical tests were performed using GraphPad, Prism version 9.2.0 for Windows, GraphPad Software, San Diego, California, USA (www.graphpad.com). Figures were subsequently compiled with PowerPoint, Microsoft Office.

## Results

### *Origin and adaptation of* P. falciparum *isolates*

**Figure 1.**
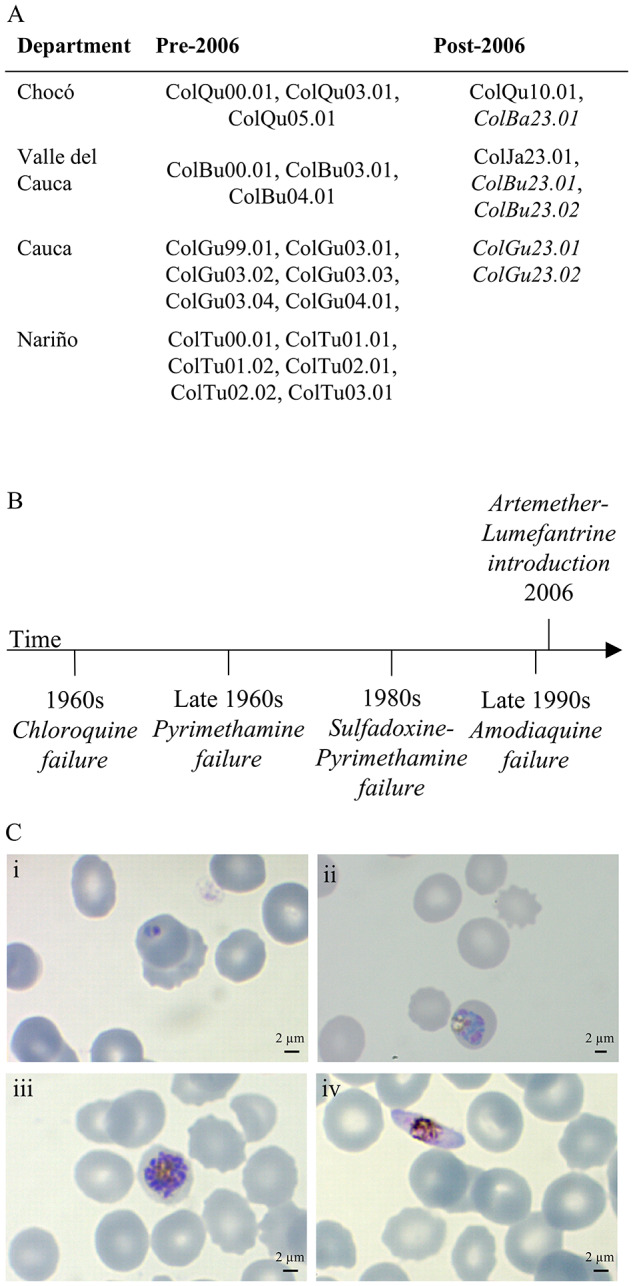
Isolates used in this study. (A) Table indicating the origin of each *Plasmodium falciparum* isolate organized according whether they were collected prior or after 2006. Those in italics failed to fully adapt to *in vitro* culture to a point where they could be characterized further. (B) Timeline illustrating the consecutive historical failure of antimalarial drugs chloroquine, pyrimethamine, sulfadoxine-pyrimethamine and amodiaquine in Colombia, and the recent introduction of artemisinin–lumefantrine in 2006, adapted from Carrasquilla *et al.,* 2022, and previous studies of sensitivity (Blair-Trujillo *et al.,* 2002). (C) Parasites at ring (i), trophozoite (ii), schizont (iii) and gametocyte (iv) stages of contemporary isolate *ColBa23.01* after introduction into *in vitro* culture. Scale bar shows 2 µm. Figure 1 long description.

*Plasmodium falciparum* isolates used in this study were collected from across the 4 departments (regional government subdivisions) of the Pacific Coast of Colombia (Figure 1A). Isolates were collected from 1999 to 2023, a period during which national antimalarial drug policy changed significantly, with the withdrawal of amodiaquine plus S-P as frontline therapy and replacement with ACTs in 2006 (Figure 1B). Given the significance of this shift for the drug pressure acting on local *P. falciparum* populations, isolates collected prior to implementation of ACT in 2006 are referred to as historical isolates and those collected after 2006 as contemporary.

Historical isolates were collected and initially adapted to local *in vitro* culture conditions (Echeverry *et al.,* 2006; Aponte *et al.,* 2011), then cryopreserved until their use in this study. These historical cryoisolates were thawed and adapted over time to synthetic serum-supplemented culture media in Cambridge, as described in Methods. The antimalarial susceptibilities of the isolates were assessed by *in vitro* drug susceptibility assays, further described under Methods, and by genotyping at loci previously associated with drug resistance (both single nucleotide variants and copy number variants).

Contemporary isolates were collected from patients admitted with a positive *P. falciparum* diagnosis at the *Hospital Universitario del Valle* in the period July–December 2023, and another isolate was kindly provided by the Centro Internacional de Entrenamiento e Investigaciones Médicas - CIDEIM, Cali, Colombia (ColQu10.01, collected in April 2010). These were adapted to *in vitro* culture over a 5-week period in Colombia. The antimalarial susceptibility of these isolates was characterized in the same manner as the historical isolates. Of the 7 contemporary isolates collected, 5 demonstrated some growth *in vitro* (ColBu23.01, ColGu23.02, ColBu23.02, ColJa23.01 and ColQu10.01, example shown in Figure 1C), but only 2 of these were successfully characterized (ColJa23.01 and ColQu10.01). ColBa23.01 never adapted to *in vitro* culture but instead was assessed using an *ex vivo* approach, further described under Methods. This low success rate reflects well-established challenges in the adaptations of field isolates to *in vitro* culture (Niaré *et al.,* 2018).

We subsequently obtained clinical correlates for the 5 out of the 7 patients from which the contemporary field isolates were taken, as described in Table 1. In the patient cohort, there were 3 pregnant women and 1 child under 5, which is expected given these groups tend to suffer more significant disease and are more likely to require management in a tertiary care centre, such as the *Hospital Universitario del Valle* where these isolates were collected. The low parasitaemias in patients, from which isolates ColBu23.02 and ColGu23.01 were collected, are in keeping with previous work on *P. falciparum* infections in South America (Arévalo-Herrera *et al.,* 2015), and may explain the low percentage of successful *in vitro* adaptation of isolates. All patients responded well to treatment with ACTs.

**Table 1.** Clinical data corresponding to isolates recovered. The table describes the clinical features of the patients at the Hospital Universitario del Valle from which the *P. falciparum* isolates were collected, detailing the age, sex, pregnancy status and parasitaemia at time of diagnosis. Those marked with asterisks were successfully used in *ex vivo* or *in vitro* drug susceptibility screens. N/A under parasitaemia means the data was not recorded Table 1 long description.

Isolate ID	Age	Sex	Pregnant	Parasitaemia (parasites/µL)
**ColBu23.01**	24	F	Y	N/A
**ColBu23.02**	9	M	N	2 658
ColBa23.01*	34	F	Y	28 160
**ColGu23.01**	20	F	Y	1 920
ColJa23.01*	1	F	N	360 833

### Chloroquine sensitivity

**Figure 2.**
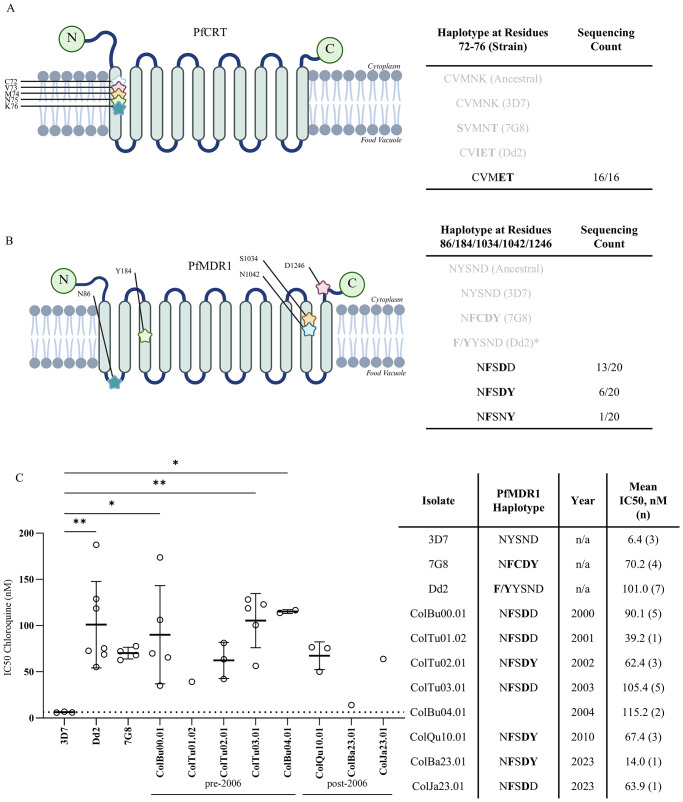
Chloroquine sensitivity genotypes and phenotypes. Stylized diagrams of *P. falciparum* chloroquine-resistance associated multipass transmembrane proteins PfCRT (A) and PfMDR1 (B), both created using Biorender.com, with orientation based on TOPCONs (Tsirigos *et al.,* 2015) prediction of transmembrane domains, annotated with the position of variants previously associated with chloroquine resistance. The respective tables to the right show the PfCRT and PfMDR1 haplotypes for *P. praefalciparum* (here denoted as ancestral), and for lab strains 3D7, 7G8 and Dd2, and the frequency of haplotypes for each transmembrane protein in the Colombian isolates tested, with mutants in bold. The asterisk highlights the Dd2 strain, which has 2 copies of PfMDR1 each with a unique variant at position 86 (Friedrich *et al.,* 2014). (C) *In vitro* chloroquine sensitivity profiles of control lab strains and Colombian field isolates. Lines and error bars show means and standard deviations, respectively. The dotted line shows the mean for negative control (3D7). Figure produced with GraphPad, Prism. The data is summarized in the adjacent table, which lists isolate ID, PfMDR1 haplotype, year of collection and mean IC50 value (nM) with number of biological replicates. Figure 2 long description.

Chloroquine resistance was first assessed by genotyping PfCRT and PfMDR1, which were successfully characterized for 16/21 and 20/21 isolates respectively. All isolates successfully genotyped contained the CVM**ET** haplotype in PfCRT (Figure 2A), which is expected given previous studies demonstrating its presence as the major haplotype in Colombia (Restrepo-Pineda *et al.,* 2008). By contrast, the isolates displayed a broader range of PfMDR1 haplotypes (Figure 2B). Most isolates contained the haplotype N**F**S**D**D (13/20), while a minority (2 contemporary and 4 historical isolates) contained triple-mutant N**F**S**DY**, namely ColBa23.01, ColQu10.01, ColQu05.01, ColTu01.01, ColQu03.01 and ColTu02.01. Only isolate ColTu00.01 had the double-mutant N**F**SN**Y** haplotype (1/20). The presence of C-terminal variants in this region (S1034C, N1042D and D1246Y) agrees with wider literature, and the prevalence of N**F**S**D**D as the major haplotype agrees with previous studies (Echeverry *et al.,* 2007; Montenegro *et al.,* 2021).

Isolates were also characterized phenotypically for chloroquine resistance using *in vitro* IC50 assays. They demonstrated a wide range of sensitivities (Figure 2C), from contemporary isolate ColBa23.01 (IC50 = 14.0 nM, *n* = 1 *ex vivo* assay) to historical isolate ColTu03.01 (IC50 = 105.4 nM, *n* = 5 *in vitro* assay). While the IC50 average of all Colombian isolates was higher than the chloroquine sensitive control 3D7, only historical isolates ColBu04.01, ColTu03.01 and ColBu00.01 were significantly higher (ANOVA, *p* = 0.0067 and *p* = 0.0260 respectively), presumably due to variability between assays. The chloroquine resistant Southeast Asian line Dd2 was also significantly more resistant than 3D7 (ANOVA, *p* = 0.0060), validating the phenotyping methodology. The IC50 values, number of biological replicates and significances are summarized in Figure 2C.

While we have used ANOVA to assess significance, we note that the Mann–Whitney *U*-test is widely used in the field for such comparisons (Hagenah *et al.,* 2025). Using this test, Dd2 and isolates ColTu03.01 and ColBu00.01 are significantly more resistant to chloroquine than 3D7 (*p* < 0.05) (Supplementary Table 2). We favoured the ANOVA for our principal analysis given the small sample size precluding use of the Mann–Whitney *U*-test for any data sets of *n* < 2 and multiple comparisons across several datasets. We do however note that Dd2 and isolates ColTu03.01 and ColBu04.01 have IC50 values >100 nM, which has been used as a threshold for chloroquine resistance in other studies (Zhao *et al.,* 2022).

### Mefloquine sensitivity

Mefloquine sensitivity is genetically characterized by copy number variation and polymorphisms at PfMDR1 (Reed *et al.,* 2000; Price *et al.,* 2004). The same polymorphisms highlighted as mediators of chloroquine sensitivity in Figure 2B also mediate mefloquine sensitivity.

**Figure 3.**
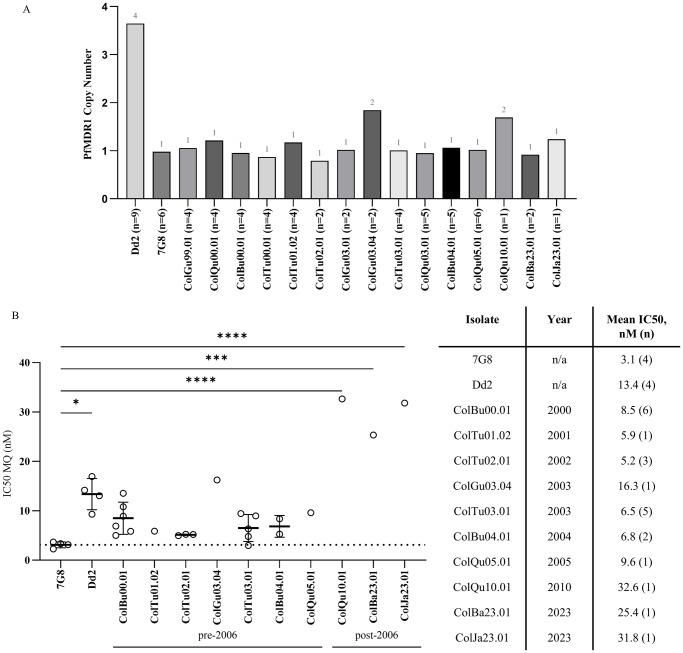
Mefloquine sensitivity genotypes and phenotypes. (A) Copy number of PfMDR1 as measured by qPCR using genomic DNA extracted from control lab strains and field isolates. The number of replicates for each assay are shown in brackets next to the isolate ID on the *x*-axis, and the mean copy number rounded to the nearest integer is shown above the bar. (B) *In vitro* mefloquine sensitivity profiles of control lab strains and field isolates. Lines and error bars show means and standard deviations for each isolate, respectively. The dotted line shows the mean for negative control 7G8. The data is summarized in the adjacent table, which lists isolate ID, year of collection and mean IC50 value (nM) with number of biological replicates. Figure 3 long description.

Copy number variation at PfMDR1 was assessed using qPCR, as described in Methods, for 15 isolates (Figure 3A). About 86.7% (13/15) of isolates had a single copy of PfMDR1, whereas historical isolate ColGu03.04 (*n* = 2) and contemporary isolate ColQu10.01 (*n* = 1) had 2 copies. About 13.3% of isolates (2/15) having more than 1 copy of PfMDR1 is in keeping with previous *in vitro* studies of isolates from Colombia (Echeverry *et al.,* 2007; Echeverry, 2013; Aponte *et al.,* 2017; Montenegro *et al.,* 2021). The PfMDR1 haplotypes are further discussed above. The dominant PfMDR1 haplotype, N**F**S**D**D, is associated with increased mefloquine resistance (Reed *et al.,* 2000), which is surprising given the absence of long-term mefloquine use in Colombia.

In concordance with the copy number variant results, the historical isolates showed a broadly low level of mefloquine resistance in the *in vitro* susceptibility assays (Figure 3B). No historical isolates were significantly more resistant than the sensitive 7G8 control. 7G8 was used here as a control of sensitivity given its favourable profile as a mefloquine sensitive control, and the failure in our hands to obtain reliable mefloquine sensitivity results with 3D7. The sample size of *in vitro* assays precludes any conclusion about the role of PfMDR1 copy number variation as a mediator of resistance in this background. The significantly increased resistance of resistant line Dd2 compared to sensitive 7G8 (ANOVA, *p* = 0.0119) validates this assay as a measure of *in vitro* mefloquine sensitivity in our hands.

Curiously, the contemporary isolates demonstrated a significantly increased mefloquine resistance compared to the historical isolates (contemporary vs historical isolates, Mann–Whitney *U*-test, *p* = 0.0167) as shown in Supplementary Figure 1. This may reflect different culturing and assaying environments as described below, an artefact of the small number of replicates for the contemporary isolates or an increase in mefloquine resistance between the 2 collection periods.

As with the analysis of chloroquine sensitivity, we compared the isolates to the negative control using the Mann–Whitney *U*-test (Supplementary Table 2.) Using this test, Dd2 and isolate ColBu00.01 were significantly more resistant to mefloquine than 7G8 (*p* < 0.05). We further note that contemporary isolates ColJa23.01 and ColQu10.01 have IC50 values >30 nM, which has been used as a threshold for resistance previously (Zhao *et al.,* 2022).

### Pyrimethamine and sulfadoxine sensitivity

**Figure 4.**
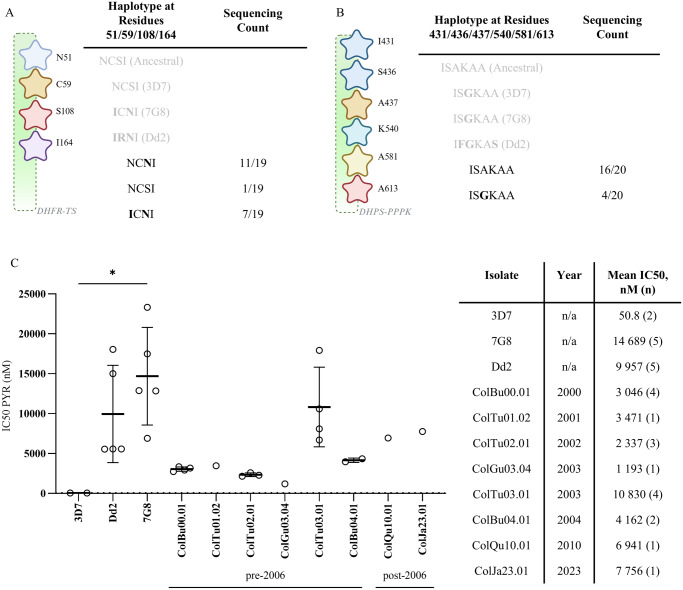
Pyrimethamine and sulfadoxine sensitivity genotypes and phenotypes. Stylized diagrams of (A) PfDHFR-TS and (B) PfDHPS-PPPK each annotated with key residues associated to antimalarial resistance. The respective tables to the right show the respective haplotypes for *P. praefalciparum* (here denoted as ancestral), and for lab strains 3D7, 7G8 and Dd2, and the frequency of haplotypes for each protein in the Colombian isolates tested, with mutants in bold. Cartoon created with Biorender.com. (C) *In vitro* pyrimethamine sensitivity profiles of control lab strains and field isolates. Lines and error bars show means and standard deviations, respectively. The dotted line shows the mean for negative control 3D7. The data is summarized in the adjacent table showing the isolate ID, year of collection and mean IC50 value (nM) with number of replicates. Figure 4 long description.

Pyrimethamine resistance was successfully characterized in 19 out of 21 isolates by sequencing PfDHFR (Figure 4A). The majority of isolates contained the single mutant haplotype NC**N**I (11/19), whilst a smaller proportion contained the double mutant haplotype, shared with 7G8, **I**C**N**I (7/19), namely historical isolates ColQu00.01, ColQu05.01, ColTu03.01 and ColQu03.01 and contemporary isolates ColBa23.01, ColQu10.01 and ColJa23.01. Only 1 isolate, ColGu03.02, contained the ancestral haplotype NCSI (1/19). *In vitro* susceptibility testing (Figure 4C) revealed a broad range of IC50 values ranging from historical isolate ColGu03.04 at 1193 nM (*n* = 1) to historical isolate ColTu03.01 at 10 830 nM (*n* = 4), as per Figure 4C. There was a significant difference between sensitive control 3D7 and resistant control 7G8 (ANOVA, *p* = 0.0119), validating the phenotyping assay. The absence of significant difference between isolates and the sensitive control may reflect a true sensitivity or may be complicated by the wide variation of specific isolates.

Given well-documented challenges in obtaining reliable results from *in vitro* sulfadoxine susceptibility assays (Wang *et al.,* 1997), we characterized sulfadoxine susceptibility only by genotyping at PfDHPS (Figure 4B) for 20 of 21 isolates. The majority of isolates (16/20) contained ancestral haplotype ISAKAA, while the remaining isolates contained the single mutant haplotype IS**G**KAA, containing A437G (4/20), namely historical isolates ColQu05.01 and ColGu03.01, and contemporary isolates ColBa23.01 and ColQu10.01. The isolates ColQu05.01, ColBa23.01 and ColQu10.01 were all collected from Chocó, Northwestern Colombia where there is an established presence of IS**G**KAA as the dominant PfDHPS haplotype (Corredor *et al.,* 2010). The 2 other isolates collected from this region expressed the ancestral ISAKAA haplotype, which is the second most prevalent haplotype in this region. The presence of PfDHPS IS**G**KAA in contemporary isolates ColBa23.01 and ColQu10.01 suggests sulfadoxine resistance associated-haplotypes are still circulating at a population level. The absence of triple-mutant PfDHFR in combination with A437G in PfDHPS means that these isolates all fall below the threshold of partial S-P resistance outlined in West African isolates (Naidoo and Roper, 2013).

The pairwise comparison of isolate sensitivities to the 3D7 negative control using the Mann–Whitney *U*-test revealed the absence of any isolates significantly more resistant to pyrimethamine than the negative control (Supplementary Table 2).

### Artemisinin sensitivity

The full propeller domain of PfKelch13 was sequenced for 3 contemporary isolates, i.e. all isolates collected after the introduction of ACTs in Colombia, and subsequent analyses showed the absence of any WHO-validated resistance markers (Global Malarial Programme, 2025).

## Discussion

The results demonstrate the continued presence of chloroquine, mefloquine, pyrimethamine and sulfadoxine resistance associated haplotypes in Colombia, however a wider variation in associated *in vitro* resistance profiles of chloroquine, mefloquine and pyrimethamine. The lack of significant concordance between phenotypic and genetic profiles may reflect a polygenic basis of resistance in this genomic background, other unique factors of the Colombian genomic background or the limitations of sensitivity assays in this study.

The continued presence of these resistance associated profiles was surprising given the absence of the active use of the associated antimalarial drugs since the introduction of artemether–lumefantrine in 2006. Populations with low transmission and clonal lineages have low levels of interspecific competition, which can permit the maintenance of alleles which are otherwise deleterious (Siddiqui *et al.,* 2021). This could account for the continued maintenance of resistance associated haplotypes, and contrasts to high transmission environments wherein such haplotypes are rapidly lost, as was seen with the loss of mutant PfCRT following the withdrawal of chloroquine in Malawi (Mita *et al.,* 2003).

Alternatively, the maintenance of resistance profiles may reflect active selection pressures. The Pacific Coast of Colombia contains a large number of informal mining settlements, which have been shown to be synonymous with counterfeit antimalarials and subtherapeutic dosing (de Santi *et al.,* 2016; Douine *et al.,* 2020). The associated paucity of good medical care may additionally lead to self-medicating with historical over-the-counter antimalarials, which has already been reported in this region (Diaz *et al.,* 2019). Furthermore, the use of chloroquine for the management of infection with *P. vivax*, and use of antifolates as antimicrobial chemotherapy, coupled with widespread availability of these medications over the counter, may lead to continued selection for resistance in this region.

Resistance may be alternatively maintained by epistatic interactions with other variants. For example, in the context of chloroquine resistance, PfAAT1 variants have been proposed to offset the fitness costs of PfCRT mutants in Southeast Asia (Amambua-Ngwa *et al.,* 2023), and variants in PfAAT1 have been described in this region (Carrasquilla *et al.,* 2022), or alternatively, the polymorphisms in the PfCRT haplotype may be evolutionarily adaptive also. It is worth noting in this context that the PfCRT resistance haplotype SVMNT, which arose independently, has also been maintained long-term in Brazil, despite chloroquine not being used as frontline antimalarial therapy under national malaria control policy for some time (de Abreu-fernandes *et al.,* 2025). Alternatively, the maintenance of SVMNT may reflect selection by atovaquone (Sá *et al.,* 2009), which has been used historically in the region (Kremsner *et al.,* 1989).

The significant increase in mefloquine resistance between historical and contemporary isolates was unexpected. Human population movements have previously been shown to result in the result in the introduction and spread of resistance haplotypes in this region (Corredor *et al.,* 2010; Yalcindag *et al.,* 2012), and the introduction of further, uncharacterized, mefloquine resistance associated variants may explain this shift.

Surveillance of sensitivities to historical antimalarials can involve either genomic surveillance approaches or *in vitro* sensitivity studies. Genomic surveillance is the least resource intensive but relies on an *a priori* understanding of the genetic basis of the resistance phenotype. By using a combination of genomic and *in vitro* sensitivity approaches, we established the presence of known mediators of resistance, whilst also more directly assaying the sensitivity phenotype. *In vitro* sensitivity assays can themselves be limited by issues with the reliable storage and culture of parasites in the endemic setting, time taken for isolates to adapt to *in vitro* culture and the variable nature of the sensitivity assays themselves. These challenges are reflected in the number and variability of the replicates we were able to obtain.

There is additionally an inherent risk of sampling bias given that only a fraction of the field isolates adapted to *in vitro* culture. The nature of antimalarial sensitivity may itself be inherently noisy; however, the challenges of assaying recently adapted parasites in 2 distinct laboratory settings will increase variability. In this study, such challenges included: the lack of availability of the same reagents in Colombia and the United Kingdom; but also variation and availability of the blood and serum used reflecting different populations of donors; the difference in laboratory equipment used to process assays; the paucity of gas mixtures meaning in Colombia parasites were often cultured using the candle jar technique; unstable power supply; and the relatively lower time the isolates collected in Colombia had to adapt to *in vitro* culture, and the associated use of *ex vivo* assays in this setting, compared to the extensive culture of isolates in the United Kingdom. These disparities may mean that isolates cultured in the United Kingdom, namely ColBu00.01, ColTu01.02, ColTu02.01, ColTu03.01, ColQu05.01, ColBu04.01 and isolates cultured in Colombia, namely ColQu10.01, ColBa23.01, ColGu03.04 and ColJa23.01, differ due to both experimental factors and intrinsic resistance.

This work has demonstrated the continued presence of chloroquine, mefloquine, pyrimethamine and sulfadoxine-resistance associated genotypes and a large variation in resistance phenotypes, in *P. falciparum* parasites circulating in the Pacific Coast in the past 23 years. We have also demonstrated the potential to conduct sensitivity studies in this region, and developed a panel of recently adapted isolates to further our understanding of polymorphisms on this genomic background. These findings argue for the judicious use of current frontline antimalarials and against the use of historical antimalarials as partner drugs along the Pacific Coast region.

## Supporting information

10.1017/S0031182026101711.sm001 Michie et al. supplementary material 1 Michie et al. supplementary material

10.1017/S0031182026101711.sm002 Michie et al. supplementary material 2 Michie et al. supplementary material

## Supplementary material

The supplementary material for this article can be found at https://doi.org/10.1017/S0031182026101711.

## Table 1 Long description

The table presents clinical data for patients from Hospital Universitario del Valle, focusing on age, sex, pregnancy status, and parasitaemia levels of P. falciparum isolates. Notably, the isolate ColJa23.01 from a 1-year-old female has the highest parasitaemia at 360,833 parasites per microliter, indicating a severe infection. In contrast, ColBu23.01 lacks parasitaemia data, which may affect the interpretation of its clinical significance. Two isolates, ColBa23.01 and ColJa23.01, were successfully used in drug susceptibility screens, suggesting their potential importance in research. The data highlights variability in parasitaemia levels across different ages and pregnancy statuses, with pregnant females showing both high and unrecorded parasitaemia levels.

Navigate back to Table 1.

## Figure 1 Long description

The image consists of three parts. The first part (A) is a table listing the origin of isolates from different departments in Colombia, categorized into pre-2006 and post-2006 collections. Departments include Chocó, Valle del Cauca, Cauca and Nariño, with specific isolate codes listed under each category. The second part (B) is a timeline illustrating the historical failure of antimalarial drugs in Colombia, including chloroquine in the 1960s, pyrimethamine in the late 1960s, sulfadoxine-pyrimethamine in the 1980s and amodiaquine in the late 1990s. The timeline also marks the introduction of artemether-lumefantrine in 2006. The third part (C) shows microscopic images of parasites at different stages: ring (i), trophozoite (ii), schizont (iii) and gametocyte (iv). Each image includes a scale bar indicating 2 micrometers.

Navigate back to Figure 1.

## Figure 2 Long description

The image consists of three parts. The first part (A) shows a diagram of the PfCRT protein with transmembrane domains and annotated variants associated with chloroquine resistance. A table lists haplotypes at residues 72 to 76 for different strains, with CVMET being present in all 16 sequenced samples. The second part (B) illustrates the PfMDR1 protein, highlighting specific variants at residues 86, 184, 1034, 1042 and 1246. A table shows haplotypes for various strains, with NFSDD being the most common in 13 out of 20 samples. The third part (C) presents a graph of IC50 concentrations for different isolates, with a table listing isolate IDs, PfMDR1 haplotypes, collection years and mean IC50 values in nanomolar. The graph includes control lab strains and Colombian field isolates, with lines and error bars indicating means and standard deviations. The dotted line represents the mean for the negative control (3D7).

Navigate back to Figure 2.

## Figure 3 Long description

The image contains two graphs and a table. The first graph (A) displays the PfMDR1 copy number for various isolates, measured by qPCR. The x-axis lists isolate IDs with the number of replicates in brackets, while the y-axis shows the PfMDR1 copy number. Most isolates have a copy number of 1, except for RKL9 with 4, ColGu03.04 with 2 and ColQu10.01 with 2. The second graph (B) illustrates mefloquine sensitivity profiles, with the x-axis showing isolate IDs and the y-axis indicating IC50 values in nanomolar. The graph includes lines and error bars representing means and standard deviations, with a dotted line for negative control 7G8. The table adjacent to the graphs lists isolate IDs, years of collection and mean IC50 values in nanomolar, along with the number of biological replicates. Isolates are categorized as pre-2006 and post-2006, with varying IC50 values.

Navigate back to Figure 3.

## Figure 4 Long description

The image contains three main sections. The first section (A) shows a stylized diagram of PfDHFR-TS with key residues N51, C59, S108 and I164. It lists haplotypes NCSI (Ancestral), NCSI (3D7), ICNI (7G8) and IRNI (Dd2) with sequencing counts: NCNI (11/19), NCSI (1/19) and ICNI (7/19). The second section (B) displays a diagram of PfDHPS-PPPK with residues S436, A437, K540, A581 and A613. It lists haplotypes ISAKAA (Ancestral), ISGKAA (3D7), ISGKGA (7G8), IFGKAS (Dd2) with sequencing counts: ISAKAA (16/20) and ISGKAA (4/20). The third section (C) is a graph showing IC50 values in nM for various isolates, with a table listing isolate ID, year and mean IC50 values. The graph includes control strains 3D7, 7G8 and Dd2 and field isolates from different years, with values ranging from 1193 nM to 10830 nM. The table provides details for each isolate, including the year of collection and mean IC50 values with the number of replicates.

Navigate back to Figure 4.
