# Supplementary material for: Antimalarial drug resistance in Plasmodium falciparum isolates from the Pacific Coast of Colombia
Source: Parasitology. 2026 Feb 13;153(4):549–60. doi: 10.1017/S0031182026101711 (PMC13244236; doi:10.1017/S0031182026101711)
Supplement: Michie et al. supplementary material 1 — Michie et al. supplementary material [file S0031182026101711sup001.docx]

**Supplementary Table 1:** Table showing the various ID codes given to the *P. falciparum* isolates used in this study. ID codes given to the isolates are a three-letter code of country of origin (Col), followed by a two letter code of their city of origin, and then a two number code of year, and a numerical value to differentiate it from other isolates collected from that city in that year. Other columns, left to right, show other associated IDs, ENA and Sanger IDs.

| **Isolate ID** | **Other Associated IDs** | **ENA ID** | **Sanger ID** |
| --- | --- | --- | --- |
| ColGu04.01 | 0409003D0 | ERR042228 | PW0001-C |
| ColTu01.01 | T9255D0 | ERR039930 | PW0002-C |
| ColBu00.01 | 05065D0 | ERR042227 | PW0003-C |
| ColTu02.01 | TU5390CULD0 | ERR042226 | PW0004-C |
| ColTu01.02 | 304303D0 | ERR042229 | PW0005-C |
| ColTu00.01 | 312703D0 | ERR042230 | PW0006-C |
| ColTu03.01 | 3113D0 | ERR042222 | PW0007-C |
| ColQu00.01 | QB1114CULD0X | ERR039903 | PW0008-C |
| ColQu05.01 | QB1147CULD0 | ERR039986 | PW0009-C |
| ColBu04.01 | 05041D0 | ERR042679 | PW0010-CW |
| ColQu03.01 | QB1266CULD0 | ERR042224 | PW0012-C |
| ColGu03.01 | 4027D0 | ERR042231 | PW0013-C |
| ColGu99.01 | 04096D0 | ERR042233 | PW0014-C |
| ColGu03.02 | 04039D0 | ERR042232 | PW0015-C |
| ColTu02.02 | T8064CULD0 | ERR039988 | PW0016-C |
| ColBu03.01 | A2698CULD0 | ERR042223 | PW0017-C |
| ColGu03.03 | 4059 |  |  |
| ColGu03.04 | 4109 |  |  |
| ColQu10.01 | QU010 |  |  |
| ColBa23.01 |  |  |  |
| ColJa23.01 |  |  |  |
| ColBu23.01 |  |  |  |
| ColGu23.02 |  |  |  |
| ColBu23.02 |  |  |  |
| ColGu23.01 |  |  |  |

**Supplementary Table 2:** Summary of p values for pairwise comparisons between isolates and negative controls using Mann Whitney U test. n.s. denotes not significant, and n<2 highlights data sets which containing <2 replicates, which precludes the use of the Mann Whitney U test.

| **Chloroquine** | |
| --- | --- |
| **Dataset compared to 3D7** | **Mann Whitney U Test p value** |
| Dd2 | 0.0167 |
| 7G8 | n.s. |
| ColTu02.01 | n.s. |
| ColTu01.02 | *n<2* |
| ColTu03.01 | 0.0357 |
| ColBu00.01 | 0.0357 |
| ColBa23.01 | n<2 |
| ColJa23.01 | n<2 |
| ColQu10.01 | n.s. |
| ColBu04.01 | n.s. |
| **Mefloquine** | |
| **Dataset compared to 7G8** | **Mann Whitney U Test p value** |
| Dd2 | 0.0286 |
| ColBu04.01 | n.s. |
| ColTu02.01 | n.s. |
| ColTu01.02 | *n<2* |
| ColTu03.01 | n.s. |
| ColBu00.01 | 0.0095 |
| ColQu05.01 | n<2 |
| ColBa23.01 | n<2 |
| ColGu03.04 | n<2 |
| ColJa23.01 | n<2 |
| ColQu10.01 | n<2 |
| **Pyrimethamine** | |
| **Dataset compared to 3D7** | **Mann Whitney U Test p value** |
| Dd2 | n.s. |
| 7G8 | n.s. |
| ColTu02.01 | n.s. |
| ColTu01.02 | n<2 |
| ColTu03.01 | n.s. |
| ColBu00.01 | n.s. |
| ColGu03.04 | n<2 |
| ColBu04.01 | n.s. |
| ColQu10.01 | n<2 |
| ColJa23.01 | n<2 |
