# Supplementary figures and images for: Antimalarial drug resistance in Plasmodium falciparum isolates from the Pacific Coast of Colombia
Source: Parasitology. 2026 Feb 13;153(4):549–60. doi: 10.1017/S0031182026101711 (PMC13244236; doi:10.1017/S0031182026101711)

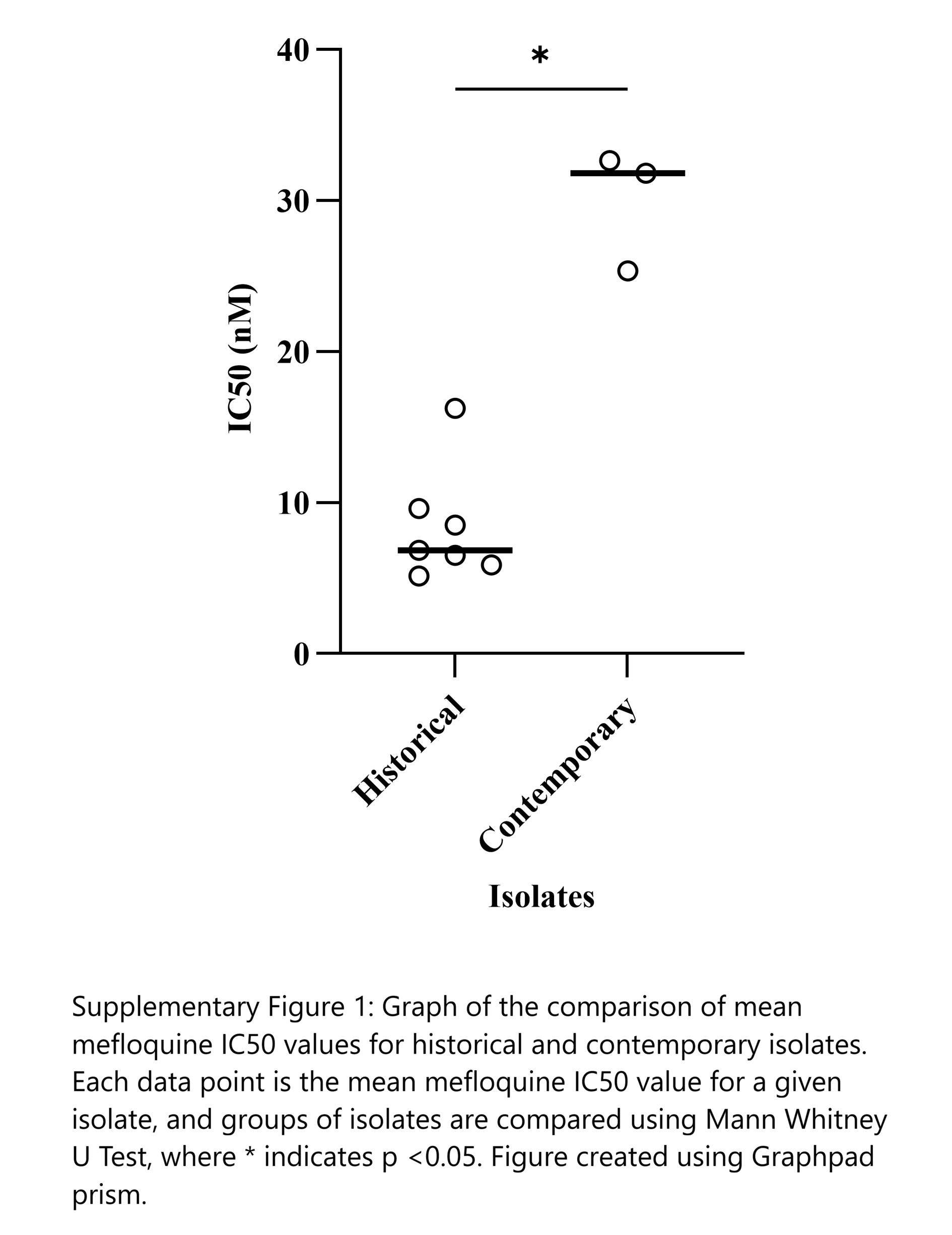

Supplement: Michie et al. supplementary material 2 — Michie et al. supplementary material [file S0031182026101711sup002.tiff]
